# Smoking, passive smoking and lung cancer cell types among women in Morocco: analysis of epidemiological profiling of 101 cases

**DOI:** 10.1186/s13104-015-1503-3

**Published:** 2015-10-03

**Authors:** Fatima Az-zahra Zakkouri, Ouaouch Saloua, Abahssain Halima, Razine Rachid, Mrabti Hind, Errihani Hassan

**Affiliations:** Department of Medical Oncology, National Institute of Oncology, Rabat, Morocco; Laboratory of Biostatistics, Epidemiology and Clinical Research, Rabat, Morocco; Department of medical oncology, National Institute of Oncology, Agdal/Riad, 10000 Rabat, Morocco

**Keywords:** Lung cancer, Women, Survival

## Abstract

**Background:**

Recently women’s lung cancer mortality rates have dramatically increased in developed countries, contrasting with a levelling off or decrease among men. Descriptive epidemiological data on primary lung cancer in women is scarce in Morocco. The aim of this study, conducted in the National Institute of Oncology in Rabat, was to describe the epidemiological profiling especially for the smoking status, to determine the most frequent type of lung cancer, and to analyse the survival of Moroccan women with lung cancer diagnosis.

**Results:**

We found 101 women among 1680 (male and female) cases of lung cancer. The never-smokers were estimated to 75 %. The proportion of adenocarcinoma among never and passive smokers was higher than that of squamous cell carcinoma (SCC) (69.4 versus 30.6 %), while among women who were smokers, the most frequent histological type was SCC (63.6 %). The Cox regression analysis showed that smoking and passive smoking were not significantly associated with survival [HR: 0.62 (95 % CI 0.31, 1.30); p = 0.19] [HR: 0.56 (95 % CI 0.29, 1.08); p = 0.08] respectively. Adenocarcinoma was significantly associated with shorter survival [HR: 1.73 (95 % CI 1.05, 2.85); p = 0.03].

**Conclusions:**

The majority Moroccan women affected by lung cancer have never smoked (75 %). Environmental exposures, genetic predisposition, hormonal factors, and viral infection may all play a role in lung cancer in this category. The relation between histological type and tobacco found in our series concurred with those reported in the literature—adenocarcinoma appears to be the most frequent cell type affecting never and passive smokers. Adenocarcinoma is significantly associated with poorer survival.

## Background

In the United States, lung cancer has been the leading cause of cancer deaths in both men and women. It’s surpassed both breast cancer and colon cancer [1]. Despite the progress made in the last years in the diagnosis and treatment of this disease, men and women die each year from lung cancer. In recent years, lung cancer mortality rates in women have dramatically increased in developed countries, contrasting with a levelling off or decrease among men [2]. In women, this dramatic increase can be explained by multiple factors. The primary cause remains tobacco, which has rapidly gained social acceptance among women after World War II [3]. In contrast, the decrease of cancer mortality in men is probably attributable to the decrease in smoking rates that began earlier in men than in women [3].

According to the Moroccan registry of cancer of Rabat of 2005, lung cancer is the leading cancer in men and comes at the eleventh place in women **[**4**]**. In Morocco, a study conducted by Lalla Salma’s foundation: “Prevention and Treatment of Cancer”, showed that over 15 years the prevalence of active smoking among elderly Moroccans was estimated at 18 %, 31.5 % of which were men and 3.3 % were women. Moreover, 41 % of the population was exposed to passive smoking [5].

Purely descriptive epidemiological data on primary lung cancer in women are scarce in Morocco. The aim of this study, conducted in the National Institute of Oncology in Rabat, was to describe the epidemiological profiling, in particular the smoking status, to determine the most frequent type of lung cancer, and to analyse the survival of Moroccan women with lung cancer diagnosis.

## Methods

A retrospective study was performed enrolling 1680 cases of lung cancer, 101 (6 %) were women diagnosed between the period of January 2004 and December 2008 at the National Institute of Oncology. All these incident cases were within the respective time period.

The study respected the ethical rules of medical research involving human subjects as stipulated by the World Medical Association in the Declaration of Helsinki. The local ethical committee of the National Institute of Oncology of Rabat also approved this study; with the consentment of patients or their families.

The hospital charts, including medical history, were reviewed and the following information was collected: age at diagnosis, smoking status, histology, stage, presenting symptoms, investigations, treatment (surgical resection, radiotherapy, chemotherapy, or no treatment) and survival.

All diagnosis were proved by anatomopathologic analysis of biopsy specimen. Non small cell lung cancer was divided into adenocarcinoma, squamous cell carcinoma and large cell carcinoma.

The descriptive analysis was made by Statistical Package for the Social Science version 13.0 (SPSS). The progression free survival (PFS) was defined as the time from the start of treatment to the first documented disease progression or death from any cause. For PFS, Kaplan–Meier curves were constructed and compared by using a long-rank test. Descriptive statistics with 95 % of confidence interval were calculated according to standard procedure.

## Results

We found 101 women among 1680 cases of lung cancer (versus 1579 men). The mean age was 55.68 years (standard deviation was 13.06).

The percentage of never-smokers was 75 %, 14 % of patients had an environmental tobacco smoke exposure (the husband or another person in the family who smoke) and 11 % reported personal smoking (Table 1). The most frequent mode of presentation was chest pain (60 %), cough (54 %), dyspnea (38 %) and hemoptysis (27 %). Proportion of non small cell lung cancer at stage IV was higher (82 %), 16 % of cases were at stage III and only 2 % were classified as stage II (Table 1).

**Table 1:** Patient characteristics

	Cases	Percentage
Smoking status		
Never smokers	76	75
Passive smokers	14	14
Smokers	11	11
Symptoms		
Dyspnea	38	38
Chest pain	61	60
Cough	55	54
Hemoptysis	27	27
Intracranial hypertension	7	6
Vena cava syndrome	1	1
Phlebitis of upper limb	1	1
Stage of non small cell lung cancer		
Stage IV	83	82
Stage III	16	16
Stage II	2	2
Stage I	0	0
Lung cancer cell type		
Adenocarcinoma	60	59
Squamous cell carcinoma	33	32
Poorly differentiated carcinoma	1	1
Small cell lung cancer	7	7
Treatment		
Palliative chemotherapy	43	43
Chemoradiation	7	7
Radiotherapy	8	8
Neoadjuvant chemotherapy before chemoradiation	2	2
Best supportive care	35	34
Brain irradiation	4	4
Surgery and radiotherapy	2	2

The proportion of adenocarcinoma among never and passive smokers was higher than that of squamous cell carcinoma (69.4 versus 30.6 %), while among women who were smokers, the most frequent histological type was squamous cell carcinoma (63.6 %).

The median follow-up was 2 months (1–24 months). The histological diagnosis was based on biopsy during bronchoscopy (80 %), as well as biopsy guided by CT scan (16 %) or thoracotomy (4 %).

Palliative chemotherapy was planned in 43 % of cases, 7 % were treated by chemoradiation, 8 % by radiotherapy, 2 % received a neoadjuvant chemotherapy before chemoradiation and 2 % were treated by surgery and radiotherapy. The best supportive care were proposed in 34 % of cases and 4 % of patients received a whole brain irradiation (Table 1).

**Fig. 1:**
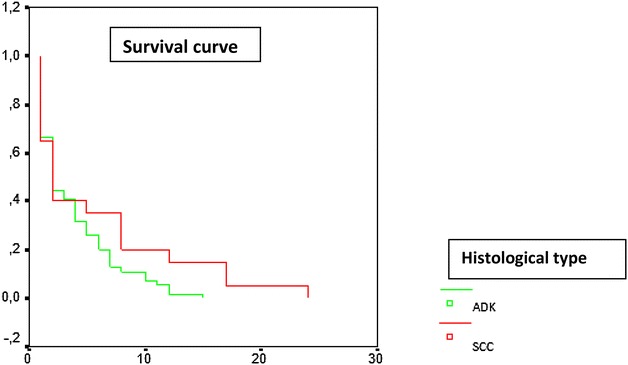
Time to death, survival curve in terms of histological type

**Fig. 2:**
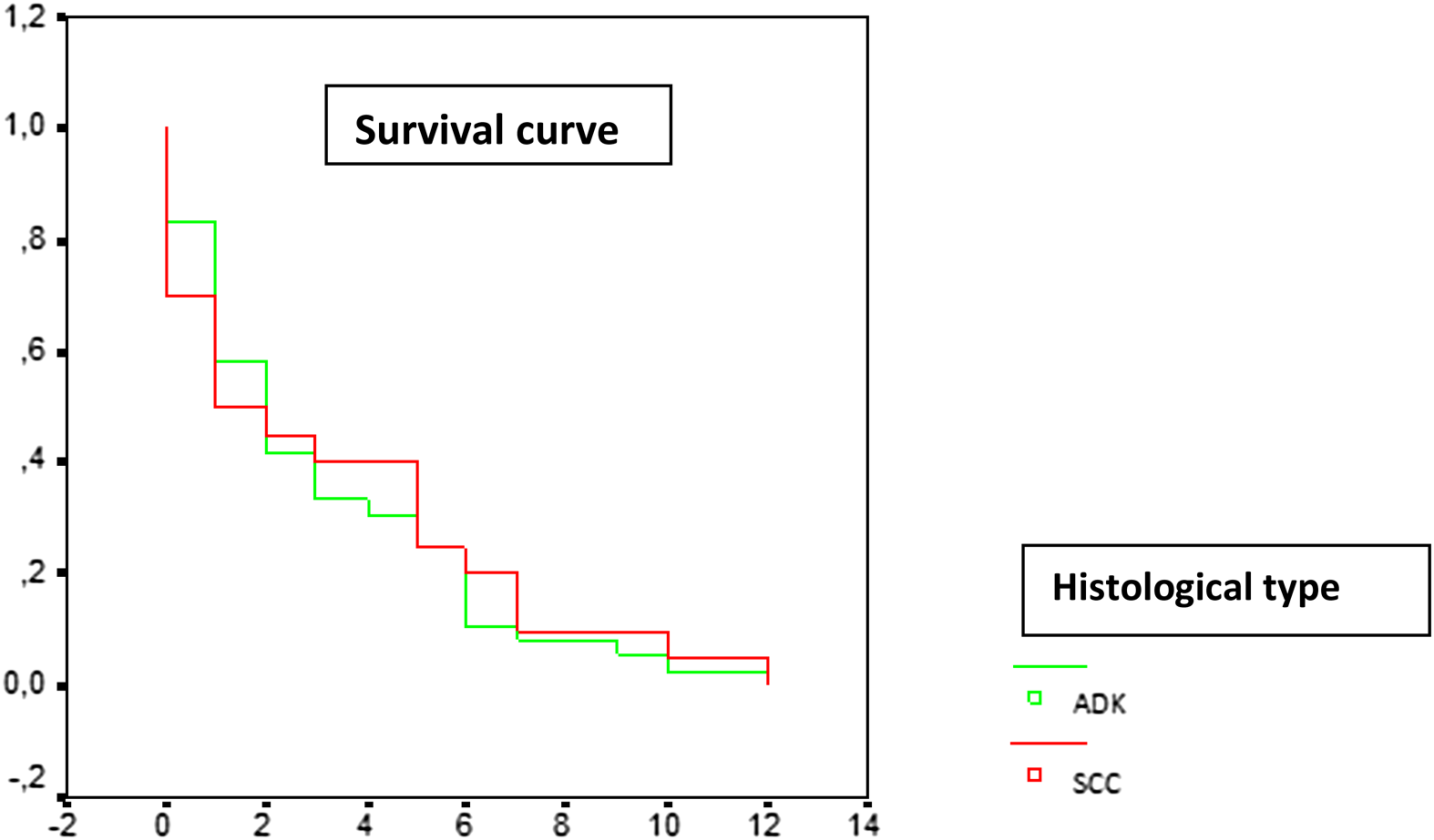
Time to progression, survival curve in terms of histological type

For follow-up cases (88 %), the survival rates of cases with no small cell lung cancer at 6 months were at 27.7 %. Cases with squamous cell carcinoma had higher survival rates than the ones with adenocarcinoma (44.4 versus 25 %; p = 0.01), but we had no significant difference in progression free survival at 6 months between squamous cell carcinoma and adenocarcinoma (20 versus 25 %; p = 0.81) (Figs. 1, 2). In cases with small cell lung cancer, the survival rate at 6 months was 0 %. 13 cases were lost to follow-up.

The Cox regression analysis showed that smoking and passive smoking were not significantly associated with survival [HR: 0.62 (95 % CI 0.31, 1.30); p = 0.19] [HR: 0.56 (95 % CI 0.29, 1.08); p = 0.08] respectively.

Adenocarcinoma was significantly associated with poorer survival [HR: 1.73 (95 % CI 1.05, 2.85); p = 0.03], brain metastasis was not significantly associated with survival [HR: 1.01 (95 % CI 0.53, 1.93); p = 0.97].

Subgroup analysis (85 patients who were never or passive smokers) showed that there was no significant difference in survival at 6 months between squamous cell carcinoma and adenocarcinoma (35 versus 20 %; p = 0.08) (Table 2).

**Table 2:** Subgroups analysis

Adenocarcinoma	Associated with survival	[HR: 1.73 (95 % CI 1.05, 2.85); p = 0.03]
Smoking	Not associated with survival	[HR: 0.62 (95 % CI 0.31, 1.30); p = 0.19]
Passive smoking	Not associated with survival	[HR: 0.56 (95 % CI 0.29, 1.08); p = 0.08]
Brain metastasis	Not associated with survival	[HR: 1.01 (95 % CI 0.53, 1.93); p = 0.97]

90.9 % (10 patients) of active smoking patients died, and one patient (accounting for 9.09 %) was lost to follow. 85 % (12 patients) of passive smoking patients died and 14.28 % (2 patients) were lost to follow.

## Discussion

The mean age was 55.68 years (standard deviation was 13.06). Our patients developed the disease at an earlier age than other women in other studies **[**6, 7**]**.

In our study, the percentage of never-smokers was 75 %. 14 % of patients had an environmental tobacco smoke exposure (the husband or another person in the family who smoke) and 11 % were reported personal smoking. In the majority of American and European reviews, most of the patients were current or ex-smokers [7–9]. Recently, approximately 23.1 % of men and 18.3 % of women in the United States have been cigarette smokers so far [10].

Tobacco does not explain all the women cases with lung cancer, because in this study, 75 % of patients were never smokers. Multiple factors such as environmental exposures, genetic mutations, hormonal factors, and some infections have also been implicated in the development of lung cancer in women who have never smoked [1]. A possible role of circulating steroid hormones particularly of oestrogens, in the etiology of lung cancer has been hypothesized [11]. It should be noted that oestrogen receptors have been detected on cancerous lung cells and estrogens induce the differentiation and maturation of the foetal lung [11–13]. Different studies have suggested that there is a potential protective effect of increased or prolonged estrogen exposure, others have found the opposite [14, 15].

Furthermore, a large meta-analysis reported a 24.5 % worldwide incidence of HPV in lung cancer, particularly in China [16] and recently, many Asian studies indicate that longer telomere length may increase lung cancer risk [17].

Studies evaluating gene environment interactions may provide important insights into carcinogenesis pathways of lung cancer in never smokers.

In European and American series, most cases of non-small cell lung cancer were diagnosed at a relatively advanced stage according to the TNM classification system [6]. In our population, proportion of non small cell lung cancer at stage IV was higher (82 %), 16 % of cases were at stage III and only 2 % were at stage II. This high percentage can be explained by the delay of diagnosis.

At the end of 1960s, changes in the frequency of different histological subtypes of lung cancer have been observed worldwide, with an increasing proportion of adenocarcinoma and a declining proportion of squamous cell carcinoma [18]. In our study, the proportion of adenocarcinoma among never and passive smokers was higher than that of squamous cell carcinoma (69.4 versus 30.6 %), while among women who were smokers, the most frequent histological type was squamous cell carcinoma (63.6 %). These results are almost similar to those reported in other studies. Adenocarcinoma represented 68.1 % in the Mayo Clinic’ study, 71 % in the Bennet’s series and 43.4 % in the Grivaux study [6, 9, 19].

Moreover, women’s 5-year relative survival rates have improved across nearly all stages with similar histologies, the benefit was more important in those with adenocarcinoma [20, 21]. In this study, adenocarcinoma was significantly associated with poorer survival [HR: 1.73 (95 % CI 1.05, 2.85); p = 0.03], while in a Radzikowska study published in *Annals of Oncology* 2002, the squamous cell carcinoma was a negative predictor of survival in univariate analysis (p = 0.008), but this result was no significant in multivariate analysis (p = 0.29) [7].

Lung cancer survival rates for non smokers have improved compared with those of smokers [22]. In this series, the Cox regression analysis showed that smoking and passive smoking were not significantly associated with survival [HR: 0.62 (95 % CI 0.31, 1.30); p = 0.19] [HR: 0.56 (95 % CI 0.29, 1.08); p = 0.08] respectively. Even though HR are neither statistically significant for smoking nor passive smoking, HR estimates seem to show a protective effect for tobacco smoking, which is highly unlikely. This may indicate some bias or confounding within the study population. However, the data does not allow additional analysis to understand these estimates.

This study had potential limitations, one of which was the retrospective nature of the analysis. Definitely, a case control study with healthy controls would have been methodologically more powerful.

## Conclusions

Many Moroccan women affected by lung cancer have never smoked (75 %). The relationships between histological type and tobacco found in our series are in agreement with those reported in the literature: adenocarcinoma appears to be the most frequent cell type among never and passive smokers. Furthermore, adenocarcinoma is significantly associated with poorer survival.

Smoking does not explain all cases of lung cancer. Environmental exposures, genetic predisposition, hormonal factors, and viral infection may all play a role in lung cancer in women.

## Abbreviations

HR: hazard ratio
CT: computed tomography
TNM: tumor, nodes and metastasis

## References

[1] Kligerman Seth, White Charles (2011). Epidemiology of lung cancer in women: risk factors, survival, and screening. AJR.

[2] Rivera MP, Stover DE (2004). Gender and lung cancer. Clin Chest Med.

[3] Jemal A, Thun MJ, Ries LA (2008). Annual report to the nation on the status of cancer, 1975–2005, featuring trends in lung cancer, tobacco use, and tobacco control. J Natl Cancer Inst.

[4] Benjaafar N, Tazi MA, Er-Raki A, et al. Moroccan Registry of Cancer. 2005:24–25.

[5] Official website of the Foundation “Lalla Salma/prevention and treatment of cancer”. 2015. http://www.contrelecancer.ma/fr/.

[6] Grivaux M, Breton JL, Bombaron P, Kuntz P, Lebas FX, Mehdaoui A, Herman D, David P, Berruchon J, Delclaux B, Zureik M, Blanchon F (2004). Lung cancer among women in France. Analysis of the 904 French women with lung cancer included in the KBP-2000-CPHG study of the French College of General Hospital-based Pneumologists (CPHG). Lung Cancer.

[7] Radzikowska E, Glaz P, Roszkowski K (2002). Lung cancer in women: age, smoking, histology, performance status, stage, initial treatment and survival. Population-based study of 20561 cases. Ann Oncol.

[8] Rachtan J (2002). Smoking, passive smoking and lung cancer cell types among women in Poland. Lung Cancer.

[9] de Andrade M, Ebbert JO, Wampfler JA, Miller DL, Marks RS, Croghan GA, Jatoi A, Finke EE, Sellers TA, Yang P (2004). Environmental tobacco smoke exposure in women with lung cancer. Lung Cancer.

[10] Lloyd-Jones D, Adams RJ, Brown TM, American Heart Association Statistics Committee and Stroke Statistics Subcommittee (2010). Heart disease and stroke statistics—2010 update: a report from the American Heart Association. Circulation.

[11] Dresler CM, Fratelli C, Babb J, Everley L, Evans AA, Clapper ML (2000). Gender differences in genetic susceptibility for lung cancer. Lung Cancer.

[12] Cagle PT, Mody DR, Schwartz MR (1990). Estrogen and progesterone receptors in bronchogenic carcinoma. Cancer Res.

[13] Mollerup S, Jorgensen K, Berge G, Haugen A (2002). Expression of estrogen receptors alpha and beta in human lung tissue and cell lines. Lung Cancer.

[14] Weiss JM, Lacey JV, Shu XO (2008). Menstrual and reproductive factors in association with lung cancer in female lifetime nonsmokers. Am J Epidemiol.

[15] Dougherty SM, Mazhawidza W, Bohn AR (2006). Gender difference in the activity but not expression of estrogen receptors alpha and beta in human lung adenocarcinoma cells. Endocr Relat Cancer.

[16] Klein F, Amin Kotb WF, Petersen I (2009). Incidence of human papilloma virus in lung cancer. Lung Cancer.

[17] Machiela MJ, Hsiung CA, Shu XO, Seow WJ, et al. Genetic variants associated with longer telomere length are associated with increased lung cancer risk among never-smoking women in Asia: a report from the female lung cancer consortium in Asia. Int J Cancer. 2015;137(2):311–9. 10.1002/ijc.29393 PMC4733320 25516442

[18] Alberg AJ, Ford JG, Samet JM (2007). Epidemiology of lung cancer: ACCP evidence-based clinical practice guidelines (2nd edition). Chest.

[19] Bennett WP, Alavanja MCR, Blomeke B, Vahakangas KH, Castren K, Welsh JA, Bowman ED, Khan MA, Flieder DB, Harris CC. Environmental tobacco smoke, genetic susceptibility, and risk of lung cancer in never-smoking women. J Natl Cancer Inst 1999;91(23):2009–14. 10.1093/jnci/91.23.2009 10580025

[20] Altekruse SF, Kosary CL, Krapcho M, et al, editors. SEER Cancer Statistics Review, 1975–2007. Bethesda: National Cancer Institute. http://seer.cancer.gov/csr/1975_2007. Based on November 2009 SEER data submission. Posted to the SEER Website 2010.

[21] Ries LAG, Young JL, Keel GE, Eisner MP, Lin YD, Horner M-J, editors. SEER survival monograph: cancer survival among adults: U.S. SEER program, 1988–2001: patient and tumor characteristics. NIH Publication no. 07-6215. Bethesda: National Cancer Institute; 2007.

[22] Kligerman S, Abbott G (2010). A radiologic review of the new TNM classification for lung cancer. AJR.

